# Continuing the Journey: The Second Edition of the Neurotrauma Treatment Simulation Center (NTSC)

**DOI:** 10.25122/jml-2023-1022

**Published:** 2023-06

**Authors:** Diana Chira, Dafin Muresanu

**Affiliations:** 1 RoNeuro Institute for Neurological Research and Diagnostic, Cluj-Napoca, Romania; 2 Department of Neuroscience, Iuliu Hatieganu University of Medicine and Pharmacy, Cluj-Napoca, Romania

## INTRODUCTION

**Figure 1:**
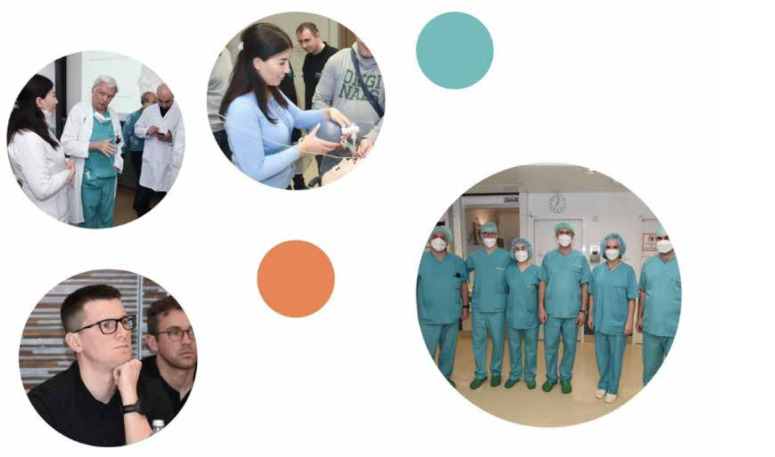
Participants and faculty from the Neurotrauma Treatment Simulation Center 2023

The Neurotrauma Treatment Simulation Center (NTSC) is an innovative educational program, aimed at bridging the gaps among specialties and promoting a multidisciplinary treatment concept to neurotrauma care. In this event report, we highlight the achievements of the second edition of the Neurotrauma Treatment Simulation Center (NTSC). The program was built upon the success of its inaugural edition which brought together medical professionals from diverse specialties (e.g., neurology, neurosurgery, neurocritical care, and anesthesia) and countries. This year’s course was held in Vienna, Austria, between the 16^th^ and 21^st^ of April and welcomed participants from Vietnam, Thailand, Ukraine, South Korea, Bosnia, and Azerbaijan who collaborated toward improving the neurotrauma patient’s pathway through the medical system and revolutionizing multidisciplinary neurotrauma management (Figure 1).

A critical focus of NTSC in neurotraumatology is shifting from the usual short-term treatment perspective toward long-term follow-up care. Creating this paradigm shift addresses the limitations of current treatment concepts and emphasizes the need for collaboration among medical professionals across the entire chain of recovery. By developing a shared understanding of the complex treatment and rehabilitation needs, the NTSC aims to enhance the quality of care for patients with traumatic brain injury (TBI).

One of the key features of the NTSC is its simulation center in Klinik Floridsdorf, which provides participants with a unique opportunity to refine their skills in a controlled environment. The simulation scenarios recreate emergency situations, enabling participants to enhance their knowledge of neurotrauma treatment complexities while practicing their collaborative skills under pressure. The inaugural NTSC-Vienna 2022 as well as NTSC-Vienna 2023 stressed the importance of multidisciplinary perspectives and experiences in developing effective treatment plans for patients with TBI.

This program has been organized by The Academy for Multidisciplinary Neurotraumatology (AMN), a forward-looking scientific organization dedicated to advancing research, treatment, and education in the field of neurotraumatology and it is endorsed by academic partners, including the European Federation of Neurorehabilitation Societies (EFNR) and other important stakeholders. By fostering strong ties with these esteemed organizations, the AMN is able to leverage shared expertise, resources, and networks, resulting in a collective effort to advance the field of neurotrauma.

**Figure 2:**
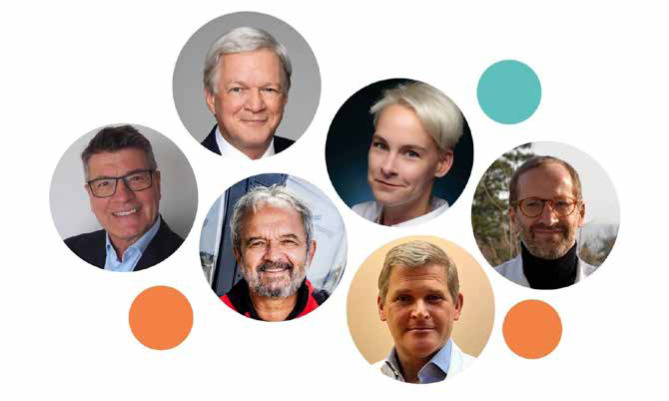
Faculty from the Neurotrauma Treatment Simulation Center 2023: Christian Matula (upper left), Andreas Winkler (lower left), Vera Wohlgennant (upper middle), Helmut Trimmel (lower middle), Peter Lackner (upper right), Johannes Leitgeb (lower right)

The five-day training program was developed and coordinated by a team of distinguished faculty members (Figure 2):

- Christian Matula – Professor of Neurosurgery, Allgemeines Krankenhaus Wien (AKH), Vienna, Austria; AMN Chairman of the Educational Committee
- Johannes Leitgeb – Associate Professor of Trauma Surgery, Allgemeines Krankenhaus Wien (AKH), Vienna, Austria;
- Andreas Winkler – Medical Director of the Institute Neuromed, Center for Clinic Sciences in Neurology, Vienna, Austria;
- Vera Wohlgennant – Deputy Medical Director, AUVA Rehabilitationszentrum Meidling, Vienna, Austria;
- Peter Lackner – Head of the Department of Neurology at Klinik Floridsdorf, Vienna, Austria;
- Helmut Trimmel – Professor, Director of the Department Anaesthesiology, Emergency and Critical Care Medicine at Landesklinikum Wiener Neustadt, Vienna, Austria.

As an integral component of the program and for the continued advancement of similar initiatives, the AMN representatives interviewed some of the NTSC program coordinators. During the interviews, the faculty members discussed their motivation for spearheading the NTSC training, obstacles encountered in neurotrauma management, discrepancies in neurotrauma practice, and the crucial role of guidelines, including the guidelines that the AMN is currently formulating.

**Figure 3:**
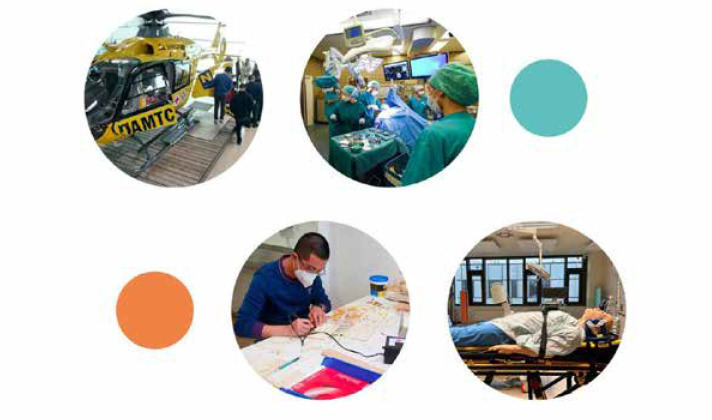
Pictures from the Landesklinikum Wiener Neustadt (top left), Allgemeines Krankenhaus Wien (top right), AUVA Rehabilitationszentrum Meidling (bottom left), and the Simulation Center at Klinik Floridsdorf (bottom right)

Drawing from their professional experience and expertise, the faculty members provided didactic sessions on neurotrauma management and investigation approaches, highlighting the value of multidisciplinary care and the necessity of long-term, ongoing patient monitoring. The training curriculum was conducted over five days at the following locations (Figure 3):

- Landesklinikum Wiener Neustadt on Day 1;
- Klinik Floridsdorf (Simulation Center) on Day 2;
- Allgemeines Krankenhaus Wien (AKH) on Day 3 and 5;
- AUVA Rehabilitationszentrum Meidling on Day 4.

## DAY 1

The first day of the NTSC started at Landesklinikum Wiener Neustadt, a teaching hospital affiliated with the Medical Universities of Vienna and Graz, and the University of Applied Sciences Wiener Neustadt. The airfield of the hospital serves as the operational base for the air rescue center.

The first day of the training included engaging presentations, starting with the ‘*Air rescue system for primary and secondary missions*’ by Dr. Daniel Csomor which showcased an overview of prehospital emergency medicine, specifically focusing on emergency call procedures in Austria, the dispatch center, and the overall emergency services and infrastructure. In further detail, Dr. Csomor delved into the composition of an air rescue team, consisting of three crucial members, namely the pilot, the emergency physician, and the paramedic, emphasizing the distinct responsibilities of the latter two within this team structure. In addition, Dr. Csomor highlighted the overarching vision of the Air Rescue Crew, which is to ensure comprehensive air rescue coverage for all individuals within their operational areas, regardless of circumstances, as well as their commitment to professionalism and unwavering passion for air rescue which forme the cornerstone of their mission.

In the next presentation entitled ‘*Pre-hospital medical care in traumatic brain injury*’, Dr. Guenther Herzer outlined the responsibilities undertaken by emergency physicians, from initial assessment to recommendations for management and diagnostics. Dr. Herzer emphasized the crucial nature of providing definitive treatment to patients with traumatic brain injuries, highlighting that the process commences and significantly influences the patient's outcome right at the accident scene. The key objectives encompass the following:

- Assessment of the patient's level of consciousness and evaluation of the pattern and severity of the injury.
- Ensuring the stability of vital functions, including breathing and circulation.
- Proper stabilization of the cervical spine to prevent further damage.
- Timely management of life-threatening associated injuries.
- Appropriately selecting the most suitable hospital for the patient's condition.
- Safe transportation of the patient to the designated hospital, along with meticulous documentation of the entire process.

Dr. Herzer emphasized the critical role of emergency physicians in ensuring prompt and effective interventions, thereby maximizing the chances of positive patient outcomes.

Following the informative presentations, the participants had the opportunity to visit the airshed, where they explored and engaged in discussions regarding the rescue mission equipment. Dr. Csomor guided the tour and shared insights about the structure of the rescue team, detailing the specific roles and responsibilities of team members during a rescue operation. Additionally, Dr. Csomor provided a comprehensive overview of the equipment used during both summer and winter rescue missions, ensuring a thorough understanding of the tools and resources available to the team in various scenarios.

In the latter part of the day, the participants and speakers met for the official opening of the five-day training program. During this session, the faculty members presented the overarching objectives and plans for the week, setting the tone for the training program and outlining the key areas of focus.

Prof. Helmut Trimmel initiated the discussions by emphasizing the crucial significance of transporting the patient to the appropriate hospital and highlighted the importance of administering neuroprotective medication as early as possible. The main message conveyed was that it is never too early to initiate the process of repairing and safeguarding the neurological well-being of the patient. Further on, Prof. Christian Matula discussed the objectives and goals of NTSC Vienna, highlighting the treatment paradigm shift from short-term focus to long-term follow-up, to ensure comprehensive care for patients throughout their recovery journey. Finally, all program tutors extended a warm welcome to the participants and provided a concise presentation about their respective days of training.

## DAY 2

The second day took place at Klinik Floridsdorf, where Prof. Peter Lackner offered the participants a captivating glimpse into one of the most sophisticated simulation centers globally. This cutting-edge facility boasts advanced technology, including complex mannequins capable of reacting to stimuli, as well as top of the line video and audio monitoring room. This advanced setup allows for realistic and immersive training experiences, enabling the enhancement of skills and knowledge in a dynamic and hands-on manner.

Prof. Peter Lackner shared an insightful presentation focused on *Multidisciplinary treatment concepts for traumatic brain injury (TBI)*. He discussed the importance of an integrated approach to TBI treatment, with emphasis on maintaining optimal cardiovascular, respiratory, and metabolic conditions while avoiding or aggressively addressing hypotension, hypoxia, and hyperthermia. The key messages conveyed during the presentation were as follows:

- Multidisciplinary treatment is essential for patients with traumatic brain injury.
- Care pathways play a vital role in connecting individuals to appropriate services and improving the overall management of TBI patients.
- Registries provide a structured framework for optimal patient management, enabling knowledge transfer and facilitating benchmarking among trauma centers.
- Simulation-based teaching courses are ideal for imparting multidisciplinary knowledge and promoting a comprehensive understanding of the entire chain of TBI care.

Prof. Lackner's presentation underscored the significance of a coordinated and comprehensive approach to treating traumatic brain injuries, emphasizing the importance of collaboration, structured pathways, registries, and simulation-based training to enhance patient outcomes.

The highlight of the day was an engaging simulation exercise involving neurotrauma cases. Participants actively collaborated to stabilize and treat a patient portrayed by a highly realistic mannequin. The activity aimed to provide practical experience in emergency scenarios and equip participants with the skills to handle challenging situations, both as leaders and team members. The training underlined the importance of effective communication within the team and maintaining smooth coordination among members, to enhance participants' abilities to work cohesively and ensure seamless functioning during critical moments. By actively engaging in this simulation exercise, participants gained valuable insights into the practical application of their knowledge and skills in a realistic and dynamic environment. To ensure the seamless execution of the day's activities, the participants were divided into two groups. One group actively participated in simulation training, while the other group observed the unfolding scenarios closely via a video feed. This arrangement enabled them to engage in discussions with specialists and experts, facilitating a comprehensive learning experience. The groups later swapped roles, allowing for a well-rounded interactive approach. By adopting this method, the training aimed to optimize the participants' exposure to diverse perspectives and promote valuable discussions, ultimately enhancing their knowledge and proficiency in managing neurotrauma cases.

The day culminated with a captivating guided tour, expertly led by Prof. Lackner, granting participants an insightful glimpse into the hospital's Stroke Unit and Neurological Ward. During the tour, attendees had the unique opportunity to witness firsthand the specialized care and treatment delivered to patients suffering from strokes and neurological conditions.

## DAY 3

The third day of the NTSC was held at Allgemeines Krankenhaus Wien (AKH) - Vienna General Hospital commencing with a concise overview of the hospital's care unit, presented by Prof. Christian Matula. The subsequent presentation shifted toward Neurotrauma Management at the University Hospital of Vienna, offering three distinct perspectives:

- *the anesthesiologist's viewpoint* (Prof. Klaus Klein)
- *the traumatologist’s viewpoint* (Prof. Johannes Leitgeb)
- *the neurosurgeon’s viewpoint* (Prof. Christian Matula).

These three approaches collectively provided a comprehensive understanding of the multidisciplinary approach employed at the University Hospital of Vienna for effective neurotrauma management.

Subsequently, the participants were divided into three groups, affording them the opportunity to visit critical areas of neurotrauma management: the operating room (OR), the endovascular interoperative magnetic resonance imaging (MRI), and the intensive care unit (ICU). These visits provided firsthand insights into the specific procedures and cutting-edge technologies utilized in neurotrauma management. The OR visit provided a glimpse into the surgical interventions and techniques used in treating neurotrauma cases. The endovascular interoperative MRI showcased advanced imaging technologies utilized during neurosurgical procedures. Lastly, the ICU visit offered participants the opportunity to observe the specialized care provided to patients recovering from neurotrauma, emphasizing the importance of intensive monitoring and support. By organizing participants into smaller groups and facilitating rotation through these different areas, the activity effectively fostered a comprehensive and immersive learning experience. This approach enabled participants to witness various facets of neurotrauma management firsthand and attain a deeper understanding of each stage of patient care.

Later in the day, participants had the opportunity to engage in interactive discussions about real-life neurotrauma cases with specialists. By collaborating with the hospital doctors, the participants could delve into the complexities of each case, share their insights, learn from the expertise of the medical professionals, and gain a deeper understanding of the decision-making process and the various factors to be considered in neurotrauma management.

## DAY 4

The fourth day of NTSC took place at AUVA Rehabilitationszentrum Meidling, a renowned rehabilitation center in Meidling, Vienna. Specializing in neurotrauma rehabilitation, the center offers comprehensive, personalized, and patient-centered care to support recovery and improve quality of life.

Dr. Vera Wohlgennant delivered a presentation on *TBI rehabilitation in Austria*, emphasizing the significance of early rehabilitation intervention and the essential components of TBI rehabilitation, such as a robust rehabilitation infrastructure, the involvement of specialized multidisciplinary teams, and the focus on restoring the best possible individual functional outcome. Dr. Wohlgennant also underscored the significance of comprehensive rehabilitation in helping patients regain their independence during the rehabilitation process and optimizing outcomes for individuals with TBI.

Dr. Murg-Ageny discussed *Neurorehabilitative aspects in the early stages of traumatic brain injury (TBI)* focusing on the significance of clinical findings, imaging techniques, and various diagnostic tools such as somatosensory evoked potentials (SSEP), transcranial Doppler (TCD), and electroencephalogram (EEG) in assessing and monitoring patients with TBI. The presentation also highlighted the importance of complication detection and prevention, as well as treatment strategies in influencing the prognosis and planning rehabilitation interventions. The key message conveyed by Dr. Murg-Ageny was the crucial role of initiating rehabilitation therapy as early as possible in the management of TBI. This emphasis on early intervention reflects the understanding that timely rehabilitation can contribute to optimizing outcomes and achieving the best possible functional recovery.

Throughout the day, the participants attended presentations covering essential topics concerning traumatic brain injury rehabilitation. These sessions delved into critical aspects like swallowing and language function. Notably, a demonstration of the implementation of a nasogastric tube for dysphagia rehabilitation was also featured, providing valuable insights into the practice.

Additionally, the faculty discussed neuropsychological TBI rehabilitation, emphasizing the psychological and cognitive aspects of recovery, the role of physiotherapeutic strategies in early rehabilitation to promote functional improvement, and occupational therapy and orthopic treatments, with a focus on enhancing daily living skills and addressing musculoskeletal challenges in TBI rehabilitation. These presentations collectively provided a comprehensive understanding of the multidisciplinary nature of TBI rehabilitation and the diverse strategies employed to support patients throughout their recovery.

## DAY 5

The last day of NTSC took place at Allgemeines Krankenhaus Wien (AKH) - Vienna General Hospital. The participants' implementation plans, developed over the course of the week, stood as manifest to their creative thinking and innovative approaches in addressing the gaps in neurotrauma management within their respective home countries. By leveraging their newfound expertise and assimilating the insights gained during the training, these plans were strategically designed as comprehensive roadmaps. With these well-structured plans in place, participants are poised to enhance their healthcare systems and effectively implement strategies that will ultimately lead to improved patient outcomesThe NTSC program not only fostered knowledge exchange but also empowered the participants to take tangible steps toward advancing neurotrauma care in their regions.

Additionally, Dr. Agata Andrzejewska (Poland), a participant in the 2022 program, returned to Vienna for this year's program and delivered updates on their country's implementation plan, showcasing the progress made since the previous year's training. Dr. Andrzejewska's presentation emphasized the significance of engaging even the most resistant individuals, as this would pave the way for gathering support from the entire team and highlighted the importance of unity and her team’s initial focus on engaging the neurosurgeon, recognizing their pivotal role in driving transformative change. Another crucial point of the presentation was the adoption of the 5Ps framework: plan, patient-centered care, protocols, preparation, and practice. Acknowledging the existing challenges, the talk embraced a proactive stance, emphasizing the collective understanding that improvement could be attainable. In her message to the 2023 NTSC participants, Dr. Andrzejewska highlighted the practical application of the knowledge and skills gained in the previous year offering inspiration for this year’s attendees to pursue ambitious goals.

The day's proceedings culminated in extensive discussions centered around the pivotal takeaways from the five-day program. Participants engaged in reflective conversations, openly sharing their valuable insights, drawing significant conclusions, and offering constructive feedback. These discussions served as a platform for evaluating the program's overall effectiveness, identifying areas for enhancement, and capturing diverse perspectives on the experience. The feedback gained during these discussions will play a pivotal role in refining future NTSC-Vienna programs, ensuring their continued success in fostering collaboration, facilitating robust knowledge exchange, and driving advancements in the field of neurotrauma treatment.

## CONCLUSION

The Neurotrauma Treatment Simulation Center (NTSC) program plays a pivotal role in advancing the knowledge and skills of healthcare specialists involved in the comprehensive treatment chain of neurotrauma. Recognizing the complex nature of neurotrauma cases, NTSC is dedicated to promoting multidisciplinary collaboration and providing a platform for experiential learning that transcends traditional boundaries.

**Figure 4:**
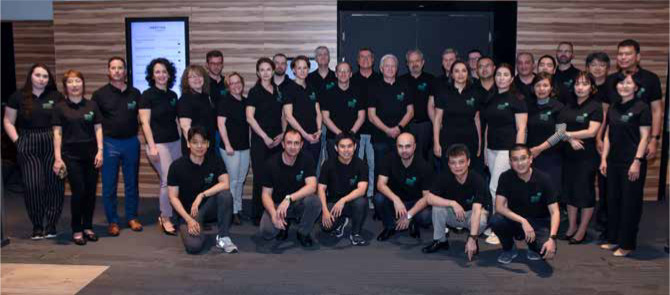
The Faculty and Participants from the Neurotrauma Treatment Simulation Center 2023

The NTSC program is brought to life through the dedicated involvement of its faculty members and participants (Figure 4). These specialists, driven by a shared passion for improving neurotrauma treatment, actively contribute their expertise, experiences, and insights to the program. Through simulation-based learning, case discussions, and knowledge exchange, the program cultivates a transformative learning environment that empowers participants to revolutionize the neurotrauma treatment paradigm.

In summary, NTSC is a dynamic and transformative initiative that brings together specialists from various disciplines to advance the understanding and treatment of neurotrauma. By promoting multidisciplinary collaboration, emphasizing long-term follow-up, and fostering transformative learning experiences, the program is poised to make a significant impact in advancing neurotrauma treatment and improving patient outcomes.

